# What does it cost to deliver antenatal care in Papua New Guinea? Results from a health system costing and budget impact analysis using cross-sectional data

**DOI:** 10.1136/bmjopen-2023-080574

**Published:** 2024-11-27

**Authors:** Olga Phoebe Martinella Saweri, Neha Batura, William Pomat, Andrew John Vallely, Virginia Wiseman, WANTAIM Study Group, Andrew Vallely

**Affiliations:** 1 Papua New Guinea Institute of Medical Research, Goroka, Papua New Guinea; 2 The Kirby Institute, University of New South Wales, Sydney, New South Wales, Australia; 3 Institute for Global Health, University College London, London, UK; 4 Health Economics, London School of Hygiene & Tropical Medicine, London, UK

**Keywords:** Maternal medicine, HEALTH ECONOMICS, Health Care Costs, Pregnancy, Pregnant Women

## Abstract

**Abstract:**

**Objective:**

In Papua New Guinea (PNG), antenatal clinic attendance averaged 50% for one or more visits, and 30% for four visits in the last decade. In 2016, the WHO revised its focused antenatal care (ANC) model recommending eight rather than four visits. If implemented, this new model would require additional resources. This study estimated provider costs of ANC in PNG, including the expected cost of scaling up to universal ANC coverage as well as recommending eight visits.

**Design and setting:**

Cross-sectional estimation of ANC costs collected from nine health facilities, which were part of a cluster randomised trial. Costs were estimated using both top-down and bottom-up approaches. The cost of the first and follow-up visits were estimated per woman, at the health facility level. Health system and scale-up costs of four visits were calculated by multiplying the aggregate cost of four visits by ANC utilisation rates. A budget impact analysis estimated the expected costs of delivering eight visits over 5 years. Univariate sensitivity analysis was conducted. Discounted costs are reported in local currency and 2019 international dollars using purchasing power parity data.

**Results:**

The average cost of the first and follow-up visits were $17.66–$30.58 (K42.94–K74.34) in Madang and $11.26–$35.61 (K27.37–K86.56) in East New Britain. Four visits per woman cost $70.65–$122.33 (K171.76–K297.36) in Madang and $45.02–$142.45 (K109.50–K346.4) in East New Britain; and salaries represented the largest share of costs. The annual health system cost was $6.9 million (K16.9 million), the expected cost of scaling up to the universal coverage of four visits was $22.7 million (K55.2 million), and $45.4 million (K110.3 million) over 5 years for eight visits.

**Conclusion:**

Costs varied with the number of clinicians, infrastructure and ANC coverage, suggesting scaling up requires increasing the financial investment in ANC services. These results provide a template to strengthen health systems by improving the quality of care.

**STRENGTHS AND LIMITATIONS OF THIS STUDY**

- This study is the first costing analysis of antenatal care (ANC) services conducted in a low- and middle-income country in the Asia-Pacific region.
- A combination of top-down and bottom-up approaches was used to derive a comprehensive costing analysis.
- The budget impact analysis will enable national health policy makers to effectively plan for the provision of quality ANC services.
- A modest number of health facilities included in the sample.
- The analysis only used the number of pregnant women attending either one or four antenatal clinic visits, as no data was available for pregnant women attending two or three clinics.

## Background

 High rates of maternal, neonatal and child morbidity and mortality remain major public health challenges for many low- and middle-income countries (LMICs). Globally, in 2017 approximately 295 000 women died from preventable causes related to pregnancy and childbirth.^1 2^ In 2015, there were 2.6 million stillbirths and 2.7 million neonatal deaths,^3 4^ the majority of which occurred in LMIC.^1^ Studies indicate that access to quality antenatal care (ANC) can help reduce maternal and neonatal mortality.^5^,^9^ Timely antenatal clinic attendance through routine care not only encourages safe, supervised deliveries at health facilities, but is a cost-effective intervention and reduces the likelihood of an adverse event during pregnancy.^2 10 11^ Further, evidence suggests that equitable access to routine ANC services implies higher rates of ANC utilisation.^12 13^

Papua New Guinea (PNG) has one of the highest estimated burdens of adverse maternal and neonatal health outcomes, including but not limited to stillbirths, preterm delivery, low birth weight or neonatal infections, in the Asia-Pacific region.^14^ Due to varying estimation methods and available data, estimates of maternal mortality range between 200 and 770 per 100 000 live births, averaging approximately 500 per 100 000 live births,^14^,^16^ while neonatal deaths are approximately 22 per 1000 live births and account for about half of the under-five child mortality.^17^ PNG also experiences relatively low ANC utilisation rates in comparison with other LMICs.^18^ ANC utilisation among pregnant women in PNG has stagnated at around 50% for at least one visit over the last 10 years,^19^ and 30% for at least four visits during the same period.^17^

Antenatal services in PNG are based on the WHO’s four-visit focused ANC (fANC) model.^20^ The model addresses shortfalls in identifying complications in pregnancy by recommending at least four standardised antenatal clinic visits throughout pregnancy, and thereafter delivering safely and under supervision at health facilities.^14^ Despite the implementation of the fANC model more than 15 years ago, the proportion of pregnant women attending antenatal clinics in PNG has not improved.^21^,^26^

In an effort to tackle adverse outcomes in pregnancy,^27^ the WHO increased the recommended number of antenatal clinic visits to eight in 2016.^28^ The more often pregnant women make contact with a trained health worker, the greater the likelihood that any complications in pregnancy will be picked up, potentially reducing the risk of adverse outcomes, including neonatal infections, and neonatal and/or maternal deaths.^14^ The question of whether countries such as PNG should try and mobilise the resources necessary to double the number of antenatal clinic visits is widely debated.^27^ Mobilising resources to increase the number of recommended visits may not automatically lead to improved health outcomes.^12^ There is mixed evidence on the effectiveness of increasing the number of antenatal clinic visits to eight throughout pregnancy.^29^ Evidence suggests that improving the quality and reducing the inequity of access to ANC services may have a greater impact on adverse pregnancy and birth outcomes.^10 13^ These discussions must be informed by evidence on the cost implications of adopting the new guidelines.^30^ Given that over half of maternal deaths are linked to pre-existing conditions, increasing the number of antenatal clinic visits throughout pregnancy ensures fewer pre-existing conditions are overlooked or missed.^31 32^ This can contribute to fewer adverse outcomes throughout the perinatal period.^28^ This study aims to determine the health system costs of delivering ANC services in PNG at a provincial level. Specifically, we calculate the health system cost of delivering the fANC model (four visits), the additional resources required to scale up the fANC model to all pregnant women (henceforth referred to as the scale-up cost of universal ANC coverage), and the financial implications of adopting the new model that recommends all pregnant women attend eight antenatal clinic visits.

To the best of our knowledge, this is the first study to comprehensively assess the cost of ANC services in an LMIC in the Asia-Pacific region.^33^ Findings from this study will enable national health policy makers to effectively plan for the provision of quality ANC services and better understand the cost implications of major policy changes such as the introduction of eight ANC visits.

## Methods

### Study setting

**Figure 1:**
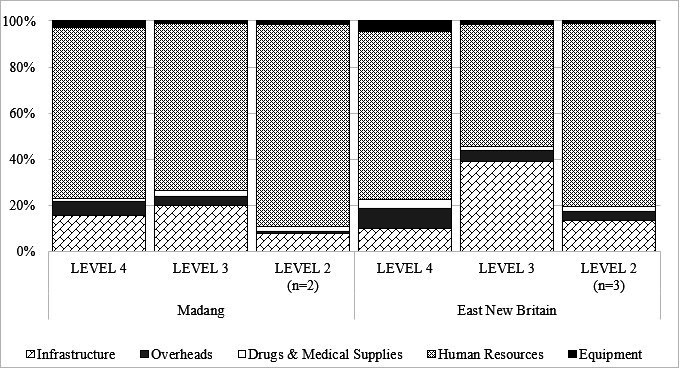
Percentage cost-breakdown of the first antenatal clinic visit for all sampled health facilities in 2019.

With a population of more than 8 million, PNG is a relatively small country in the Asia-Pacific region, however, the second largest country in the Pacific.^17 34^ PNG comprises 22 provinces in four regions and approximately 80% of the population lives in rural areas.^14 16 17 19^ Access to health services is challenging due to rugged terrain, poorly maintained buildings/infrastructure, and shortages and maldistribution of healthcare workers.^1935^,^37^ In addition, the management of the health system is guided by the policies developed by the National Department of Health, which are implemented on an ad-hoc basis at the provincial level. Therefore, the delivery of health services can differ per province. The health system is pyramidal, recently divided into six levels formerly seven, or health facility types, which distinguish primary and secondary healthcare.^38^ Primary care is provided at the first four levels, starting with community aid posts, followed by subhealth centres (and urban clinics), health centres and district/rural hospitals. Secondary care is provided at levels 5 and 6, and includes all provincial, and regional hospitals, and the only national referral hospital.^38^ The provision of maternal health services depends on the size of the health facility and the resources available,^36^ with larger health facilities tending to have greater capacity to deliver a wider range of services.^39^ The capacity for health facility deliveries, including immediate postnatal inpatient services should be available at level 3–6 health facilities, and at level 2 subhealth centres. They are not provided at level two urban clinics or level one health facilities. All operational health facilities (levels 1–6) provide outpatient services including ANC services. The PNG national ANC care package is based on the WHO model of fANC; it prescribes four antenatal clinic visits throughout pregnancy, describing their timing (and initiation) as well as the services provided.^40^ The fANC (four visits) model, also termed routine ANC, comprises two types of antenatal clinic visits: the first visit, and three follow-up visits.^35 41^ The model (see onlinesupplemental figure 1 S2), has been adapted to PNG and is detailed in the standard treatment guidelines for obstetrics and gynaecology.^41^

### Study design

This cost analysis uses data from a cluster-randomised cross-over trial in PNG: the Women and Newborn Trial of Antenatal Interventions and Management (WANTAIM) trial (ISRCTN37134032).^42^ WANTAIM evaluated the effectiveness, cost-effectiveness, health system implementation requirements and acceptability of antenatal point-of-care testing and treatment of sexually transmitted and genital infections among 4600 pregnant women and their newborns across 10 health facilities in two provinces of PNG (East New Britain and Madang). Both provinces have extensive road networks and all health facilities, including the rural health facilities, are accessible by road. The primary outcome of the trial was to determine if integrating point-of-care testing and treatment for sexually transmitted and genital infections into fANC reduces preterm birth and low birth weight compared with fANC alone.^42^

Across two provinces, nine health facilities from the trial are included in the current costing study. The sample comprises primary healthcare facilities and just over half are level two health facilities (n=5). Raw costing data collected from one health facility is incomplete, and therefore this health facility is excluded from the analysis. All health facilities in this sample are accessible by road; six are rural health facilities, two are based in provincial towns and one is in a district township. With respect to road access, six health facilities are located along a public bus route, four of which are along a main highway. The remaining four health facilities can be accessed by public transport followed by a short walk of (at most) 10 min to the antenatal clinic. Health facilities are managed either by the government or are run by a church (faith-based organisation) and employ anywhere between 8 and 30 health workers. Depending on the size of each clinic, there will be variations in the scope of services and the number of clinicians available to deliver health services including ANC. A brief overview of each type of health facility is provided in online supplemental table 1, S3.

### Costing approach

Costing data were collected from nine of the ten primary healthcare facilities participating in the WANTAIM Trial over a 6-week period between April and May 2019 by three experienced enumerators. Capital costs (eg, infrastructure and equipment) and recurrent costs (eg, human resources, medicines and medical supplies, and overheads) of ANC services were collected using a health facility assessment and costing tool specifically designed for this study.

The costs of ANC services were determined using a combination of top-down and bottom-up approaches from the healthcare provider perspective. Both methods are used to enable us to value time and resource utilisation.^43^ In this study some components of ANC are estimated using the bottom-up approach, while others are estimated using the top-down approach.^43^ Specifically, the unit costs for infrastructure, equipment and overheads are estimated using the top-down approach and the unit costs for human resources and for medicines and medical supplies are estimated using the bottom-up approach. Each component of cost is described in detail in the subsequent subsections. Enumerators collected costs directly from health facilities. In some instances, costs were collected and calculated at the provincial or national level, for example, health facility construction costs,^44^ medicines and medical supply procurement costs,^45^ and overheads, including water^46^ and electricity.^47^ Enumerators also observed the daily operation of antenatal clinics to better understand the resources used for fANC, including consultation time, and the different types of services provided. Data on health facility ANC utilisation and the number of pregnant women, were retrieved from the PNG national health indicator surveillance system, managed by the National Department of Health (see online supplemental table 2, S4).^17^

The cost of fANC, or four antenatal clinic visits, was calculated by adding the cost of the first visit and three follow-up visits (per attendee) at each health facility. This study only considers costs that are directly related to ANC and not those related to hospitalisation or patients seeking care and/or treatment unrelated to ANC. All costing equations used in this analysis are listed in online supplemental file, S5.

### Capital costs

#### Cost of infrastructure

The cost of infrastructure was estimated based on the physical space used to deliver ANC services at each health facility. The physical space used for ANC was measured by enumerators using a trundle wheel and recorded in the health facility assessment. The value per square metre for each health facility was derived by multiplying the construction cost by the physical space dedicated to ANC services. The value of this space was annualised over 30 years, which is the average lifespan of a health building in PNG,^48^ and then divided by the annual health facility ANC utilisation rate to determine the unit cost of infrastructure.

#### Cost of equipment

This cost was calculated using all equipment used during ANC and their unit prices. Enumerators listed all equipment used during clinic observations. Where possible, the procurement price of equipment was used but if this was not available, prices were obtained from common private and public-sector vendors in PNG. Health facilities also used hand-made furniture, such as beds, desks and chairs. The price of hand-made furniture was based on the amount and type of timber used and prices charged by local timber suppliers. The value of all equipment was annualised over their expected average lifespan and then divided by the annual ANC utilisation rates to determine the unit cost of equipment.

### Recurrent costs

#### Cost of human resources

The cost of human resources was estimated by determining the number of clinicians responsible for ANC services, their cadre and qualification, and their pay grade. Clinic observations were used to determine staff time (including clinic hours) and the resources used for ANC services, while staff pay grades, and salaries were obtained from health facility administration. Using staff pay grades, the annual gross (and net) salary was estimated. The number of antenatal clinics per week and the number of hours spent on ANC service-related tasks per day was used to determine the person hours and salary costs attributable to ANC services per health facility. The unit cost of human resources was calculated by dividing the annual gross salaries attributable to ANC services by annual antenatal clinic utilisation rates.

#### Cost of medicines and medical supplies

This cost was estimated by identifying the quantity and procurement prices of medicines dispensed, vaccines administered and other medical supplies used—including laboratory reagents (where applicable). Clinic observations and standard practice guidelines were used to determine the medicines and medical supplies consumed during an antenatal clinic visit. Medicines and medical supplies used in the first and follow-up visits differ resulting in two cost estimates for medicines and medical supplies. For example, HIV and syphilis testing, tetanus toxoid, mosquito nets and malaria prophylaxis were provided during first visits but not during follow-up visits. The cost of medicines and medical supplies (including vaccines) used during a consultation was derived by multiplying quantities of each drug or medical supply by the cost of a single unit.

#### Cost of overheads

Overheads attributable to ANC services were sourced from each facility and derived from administrative expenses and utilities, including electricity, water and sewage, and the use of back-up options for electricity and water. Where no payment information was available, an estimation was provided by the officer-in-charge, or staff responsible for accounting and finance. The number of clinic hours of a single antenatal clinic was used to estimate the share of overheads allocated to ANC per health facility (annualised). The annual overhead costs were divided by annual ANC utilisation rates to determine the unit cost of overheads attributable to ANC services.

### Health system cost of attending antenatal clinic four times

The annual cost of providing ANC services across PNG at government and church-run (or faith-based) health facilities was based on the average resources used to provide ANC from observed clinics and the ANC utilisation rates for each province.^49^ The average resources consumed were estimated in two steps. First, the sum of fANC was computed for each of the nine health facilities included in this analysis. Second, the average cost of fANC from all nine health facilities was calculated. The national health information surveillance system does not collect data on the number of women who attend two or three visits. Thus, the health system costs presented here relate to those women attending the currently recommended four visits in PNG. Thus, the health system costs were estimated by multiplying the number of pregnant women attending antenatal clinic four times by the average cost of four antenatal clinic visits from all nine health facilities included in this analysis.

### Scale-up costs of universal coverage of four antenatal clinic visits

Scaling-up in this analysis refers to a scenario where all pregnant women attend an antenatal clinic four times throughout pregnancy.^50^ This is defined as universal coverage of fANC. The annual expected financial cost of scaling up is calculated by multiplying the total cost of four antenatal clinic visits by the total number of pregnant women in PNG and is estimated at a provincial level.

### Budget impact analysis

The budget impact analysis (BIA) assesses the affordability of the extended model of ANC, defined as the financial investment required by GoPNG to amend national ANC guidelines to recommend that pregnant women receive ANC eight times throughout pregnancy. The aim of this BIA was to illustrate the maximum budgetary requirements for the implementation of this ANC programme across PNG over 5 years. Although 100% coverage of ANC services may not be feasible, this BIA did not model cost scenarios based on varying the utilisation of ANC services. The base case was defined as the health system cost calculated in the Health system cost of attending antenatal clinic four times section, and was compared with the expected change in financial resources over a 5-year time horizon.^51^ The BIA was based on a number of key assumptions:

1. The number of pregnant women increases year-on-year by the fertility rate, which is 3.5% annually.^52^
2. The total number of pregnant women accessing ANC is equivalent to the total number of pregnant women attending an antenatal clinic at least once.
3. The proportion of pregnant women attending an antenatal clinic at least four times is based on the average number of women attending clinic between 2019 and 2020 and remains relatively stable at 22%.^22^
4. The cost of the first and follow-up antenatal clinic visit remains fixed.

The BIA was calculated in three steps; first, the expected costs of all pregnant women attending eight antenatal clinic visits was calculated. This calculation only includes direct costs of delivering ANC services, excluding economic costs such as infrastructure and equipment. Specifically, the financial cost of making eight antenatal clinic visits was multiplied by the number of pregnant women in PNG to illustrate the budget required to deliver ANC services to all pregnant women under the new policy. Second, the budget impact was calculated, which is the difference between the base case (estimated health system cost) and the expected cost of all pregnant women attending eight antenatal clinic visits. Finally, the budget impact was forecasted, based on the aforementioned assumptions, over a 5-year period. The BIA adhered to guidelines by the International Society for Pharmaeconomics and Outcomes Research Health Sciences Policy Council.^53^

### Univariate sensitivity analysis

A univariate sensitivity analysis was conducted to account for uncertainty around the input values associated with estimating the annual health systems costs for ANC services. Table 1 describes the variables and their values used in the sensitivity analysis.

**Table 1:** Parameters and their values in the univariate sensitivity analysis

Parameter	Base case	Range	Source/assumptions
Discount rate	0.05	0.03–0.10	Based on literature^49^
ANC coverage rate	45%	±20%	Assumption based on lowest ANC coverage rate in the last 10 years and the global ANC coverage rate
Cost of infrastructure	$3.22	±20%	Based on literature^49^
Cost of equipment	$0.36	±20%	Based on literature^49^
Cost of overheads	$0.82	±20%	Based on literature^49^
Cost of human resources	$15.59	±20%	Based on literature^49^
Cost of medicines and medical supplies for the first antenatal clinic visit	$0.41	±20%	Based on literature^49^
Cost of medicines and medical supplies for a follow-up antenatal clinic visit	$0.23	±20%	Based on literature^49^

ANCantenatal care

## Data analysis

Analysis was conducted using Microsoft Excel (V.2016). All costs were collected and calculated in PNG Kina (PGK) using 2019 current prices and converted to international dollars ($) using the 2019 Purchasing Power Parity. Further, total annual health system cost of ANC services was discounted for 5%. In line with BIA guidance,^53^ the expected financial cost associated with the universal coverage of both four and eight antenatal clinic visits were not discounted. We present the results in international dollars followed by PNG Kina in parenthesis in text and tables, while only international dollars are included in the figures due to space restrictions.

### Patient and public involvement

None.

## Results

Service statistics, in ascending order of the number of pregnant women attending antenatal clinic, are presented in online supplemental table 2, S4. The table illustrates the number of pregnancies per province, and the number of pregnant women attending at least one or four antenatal clinics throughout pregnancy in 2019. From the table: Manus has the fewest number of pregnancies in 2019, yet the largest proportion of antenatal care attendees (63.8%) across the sample. In contrast, the National Capital District reported the greatest number of pregnancies; only 56% of these women attended an antenatal clinic at least once.

The cost of fANC per antenatal clinic attendee for all sampled health facilities are presented in table 2. The cost of the first antenatal clinic visit was slightly higher than that of the follow-up visits. In East New Britain, the level three health facility had the highest cost for fANC, followed by the level two health facilities, while the level four health facility had the lowest cost for fANC. In contrast, in Madang, the level four health facility incurred the greatest cost for fANC, followed by the level two health facilities, while the level three health facility incurred the lowest cost for fANC in Madang.

**Table 2:** Health facility cost of focused antenatal care services per antenatal clinic attendee in 2019 international dollars (PNG Kina)

	First visit cost	Follow-up visit cost	Sum (four visits)	Average cost
	**Range**	**Range**	**Range**	**Range**
East New Britain (n=5)				
Level 4	$11.39 (K27.76)	$11.21 (K27.24)	$45.02 (K109.50)	$11.26 (K27.37)
Level 3	$20.76 (K50.53)	$20.58 (K50.01)	$82.49 (K200.55)	$20.62 (K50.14)
Level 2(n=3)*	$19.87 (K52.11)$11.74–$35.74 (K28.61–K86.95)	$19.69 (K51.59)$11.56–$35.57 (K28.09–K86.43)	$78.95 (K206.88)$46.42–$142.45 (K112.89–K346.24)	$19.38 (K51.72)$11.61–$35.61 (K28.22–K86.56)
Madang (n=4)				
Level 4	$30.71 (K74.73)	$30.54 (K74.21)	$122.33 (K297.36)	$30.59 (K74.34)
Level 3	$17.79 (K43.33)	$17.62 (K42.81)	$70.68 (K171.76)	$17.67 (K42.94)
Level 2(n=2)*	$21.68 (K52.76)$18.59–$24.76 (K45.27–K60.26)	$21.50 (K52.25)$18.41–$24.59 (K44.75–K59.75)	$86.22 (K209.51)$73.87–$98.56 (K179.51–K239.50)	$21.55 (K52.37)$18.47–$42.64 (K44.88–K59.87)

* The average cost and the range of costs is presented where more than one facility per level was sampled.

Figure 1 illustrates the percentage cost-breakdown of the first ANC visit for all health facilities sampled. Human resources make up the greatest share of ANC costs, followed by infrastructure. In Madang, human resources make up between 72% and 88% of total costs and infrastructure between 8% and 20%. In East New Britain, human resources represent between 53% and 79% of costs and infrastructure accounts for 10%–39%. Other cost components including drugs, medical supplies and equipment, form only a small fraction of total costs.

**Figure 2:**
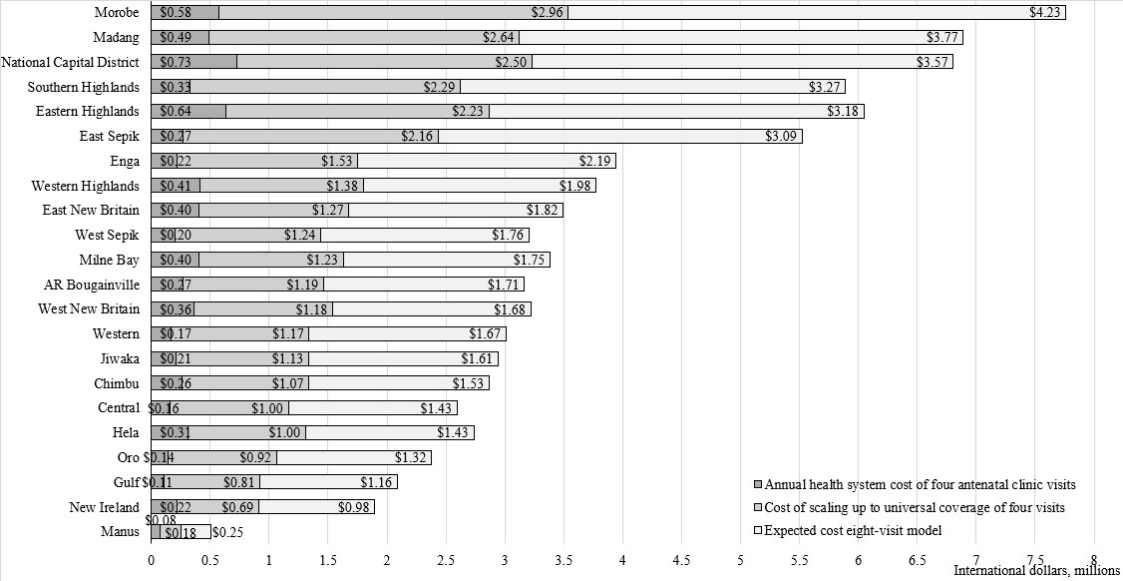
Health system cost of focused antenatal care, scaling up to universal coverage and the expected cost of the eight visit model by province in 2019 international dollars.

Figure 2 presents the annual health system costs of fANC, the cost of scaling up fANC to universal coverage and the budget impact of an eight-visit model by province. The dark grey part of the bar represents the annual health system cost, the light grey is the expected cost of scaling up fANC to universal coverage, and the white part of the bar is the expected cost of an eight-visit model. The numbers in each portion of the bar are in millions of dollars.

The annual health system cost is found by using the cost of fANC (the cost of four antenatal clinic visits) in table 2. It is calculated by multiplying the cost of fANC for all nine health facilities by the number of antenatal clinic attendees who made at least four antenatal clinic visits. The annual cost of fANC per province ranged from $0.08 million (K0.19 million) in Manus to $0.73 million (K1.76 million) in the National Capital District. The national health system cost for ANC in PNG was estimated as $6.9 million (K16.9 million).

Similarly, the expected annual financial cost of scaling up the fANC model to universal ANC coverage is found by multiplying the cost of fANC by the number of pregnant women. The expected annual financial costs of scale-up per province ranged from $0.18 million (K0.43 million) in Manus to $2.96 million (K7.2 million) in Morobe. The national annual expected cost of ANC would therefore increase from $6.9 million (K16.9 million) to $22.7 million (K55.2 million) under universal ANC coverage.

Finally, the expected cost of expanding to eight antenatal clinic visits throughout pregnancy as recommended by the 2016 WHO guidelines^28^ is estimated. The expected financial cost of the eight-visit model in 2019 is $45.4 million (K110.3) or 556% higher than the annual health system cost for fANC. East Sepik had the largest increase of $2.8 million (K6.9 million) or 1052%, which reflects a low ANC utilisation rate. In comparison, Manus had the smallest increase of $0.18 million (K0.43 million) or 226%.

**Figure 3:**
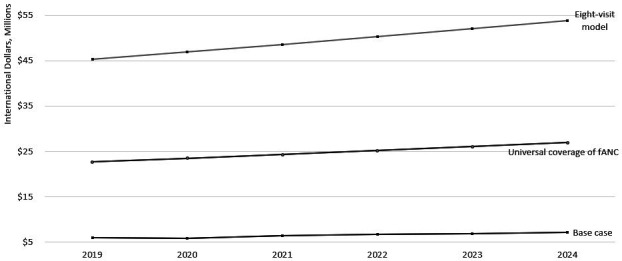
Budget impact analysis of implementing a policy recommending pregnant women receive antenatal care eight times during pregnancy in 2019 international dollars. fANC, focused antenatal care.

Figure 3 depicts the forecast of the eight-visit model over 5 years. In figure 3, the eight-visit model is compared with the base case of four visits, or the annual health system cost of fANC services and is presented in millions of international dollars. Figure 3 demonstrates that the expected cost of providing ANC would increase from $6.9 million (K16.9 million) to $45.4 million (K110.3 million) in 2019, or by 556%. The expected increase between 2019 and 2024 was $1.2 million (K2.8million) or 19.5% when assuming the current rates of ANC utilisation. We forecast that recommending eight, rather than four visits under universal ANC coverage, would increase expected annual cost from $45.4 million (K110.3 million) in 2019 by 18.8% to an expected $53.9 million (K131 million) in 2024. Between 2019 and 2024 this would equate to an average year-on-year increase of approximately $1.7 million (K4.1 million) or 3.5%.

**Figure 4:**
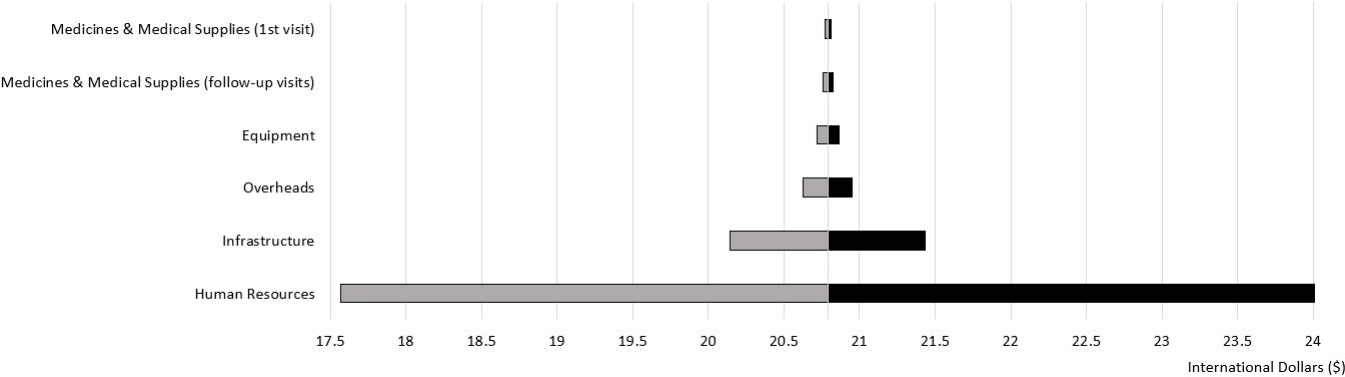
Tornado diagram: univariate sensitivity analysis of health system costs in 2019 international dollars.

The univariate sensitivity analysis was conducted to shed light on several uncertain parameters affecting the annual health system cost for ANC services. Figure 4 indicates that the results were most sensitive to changes in ANC coverage and the cost of human resources. When ANC coverage was varied by 20%, the total cost of ANC changed by $1.4 million (PGK3.4 million). Additionally, when the cost of human resources varied by 20%, the total cost of ANC changed by $1.1 million (PGK2.6 million). In terms of disaggregated costs, when the cost of human resources varied by 20%, the average cost of an antenatal clinic visit changed by $3.22 (PGK7.83). The tornado plot indicates that the cost of ANC was not very sensitive to changes in the cost of medicines and medical supplies, equipment or overheads. This is evident from the disaggregated costs, which indicate that when the cost of medicines and medical supplies were varied by 20%, the average cost of an antenatal clinic visit only changed by $0.02 (PGK0.05) for the first visit and $0.03 (PGK0.08) for follow-up visits. Further, when the cost of equipment and overheads were varied by 20%, the average cost of an antenatal clinic visit changed by $0.07 (PGK0.18) and $0.17 (PGK0.40), respectively.

## Discussion

We estimated the costs of delivering fANC services in PNG from a provider perspective using primary data from health facilities in two provinces. The total cost of four visits per woman ranged from $70.65 to $122.33 in Madang and $45.02 to $142.45 in East New Britain, with salaries and infrastructure representing the largest share of costs. This study indicated that the annual health system cost was $6.9 million (K16.9 million). The expected financial cost for scaling up to universal coverage was $22.7 million (K55.2 million), or an increase of 279%. Implementing the WHO-recommended eight-visit model would have an annual average expected financial cost of $45.4 million (K110.3 million) over 5 years, which equates to a 656% increase in the cost of delivering ANC services. When comparing the total adjusted cost of ANC services in other LMICs relative to ours; our study reported higher costs relative to studies based in Bangladesh and Uganda.^54 55^ However, our study reported comparatively lower costs than those reported in three other studies based in Ghana, Peru and Rwanda.^56^,^58^

In our study, human resources were the largest driver of ANC costs at all levels of the health system illustrating that fANC services are relatively labour intensive; that medicines and medical supplies used for ANC are relatively inexpensive as they are procured centrally via the national department of health; and equipment is not used much during visits. Our study demonstrated that the number of clinicians providing care, ANC coverage and the space allocated to provide ANC services are key drivers of cost. Comparing our results to the five earlier ANC costing studies above, two also identified human resources as being the largest share of costs,^54 56^ two listed medicines and medical supplies,^57 58^ and one listed equipment as the major share of costs.^57^ Human resources are the costliest component of ANC services where the clinical consultation is the primary focus of a antenatal clinic visit and the lab tests conducted are limited to rapid diagnostic tests. Where medicines and medical supplies or equipment are the costliest component of fANC services, either labour is relatively inexpensive, or the procurement price of medicines and medical supplies is relatively high and may include the procurement cost of equipment used in the provision of ANC services.

Scaling up fANC services to universal ANC coverage would substantially increase the cost of fANC services in PNG and is thus an important consideration for government budget allocations. Expanding budgetary allocations requires some macroeconomic growth to finance the fiscal expenditure, however, the financial investment hinges on whether ANC is a key priority area for the national government.^59^ The national budget for healthcare currently stands at $1.5 billion (PGK3.7 billion). Scaling up to universal ANC coverage of four antenatal clinic visits would increase the expected financial cost of ANC by 279%, further recommending eight antenatal clinic visits would lead to a 656% increase in ANC service costs. Both estimates would make up less than 1% of the national health budget, however, they both involve a substantial increase in costs, in part due to the underutilisation of ANC services,^56 60^ and further influenced by the fact only about 25% of pregnant women initiate ANC during their first trimester.^61^,^63^ Underutilisation and late initiation of ANC complicate the decision to scale-up or extend ANC, as there is no indication whether either is a priority, is feasible or sustainably affordable in PNG.^64 65^

Improving ANC utilisation is prioritised through the identification of determinants for utilisation, and to a greater extent treatment seeking behaviour during pregnancy. Evidence suggests that improvements in equity and quality require more than an increase in financial investment.^11^ That is, supply-side strengthening of the health system to improve the quality ANC services accompanied by demand-side interventions to encourage pregnant women to access ANC services.^66^ Both supply- and demand-side policy interventions should focus on vulnerable groups and communities to improve equity of access.^67^ Given this, exploring the relative cost-effectiveness of investing in policies, such as health education, incentive programmes or other demand-side interventions, encouraging early initiation of ANC during the first trimester of pregnancy, and their potential implications on adverse pregnancy and birth outcomes is warranted.

Our study has several limitations. First, due to a lack of country-level data pertaining to the number of pregnant women attending two or three antenatal clinic visits throughout pregnancy, estimates for health system costs only consider women who attend all four of the currently recommended antenatal clinic visits. While this underestimates the total cost of antenatal care to the health system, this estimate helps us model the costs to the health system associated with universal coverage, which can help inform decisions about scalability of services and affordability. Second, the study illustrates the maximum costs with respect to delivering ANC services on a provincial and national level, which provide a useful instrument for planning future interventions in ANC. Third, although a modest sample size may limit generalisability, the health facilities sampled capture variation in the sizes of the catchment population they serve, health services offered (and delivered), capital and material infrastructure, human resource availability and type of ownership. While the sample may be considered small, it is representative of the study area and PNG as a whole. Fourth, data availability was a challenge, ranging from a lack of receipts to limited accounting experience at some health facilities. Province level training initiatives can improve these systems; we recommend employing a routine expenditure tracking system at the health facility level to improve record keeping at health facilities and data availability. Finally, the cost-effectiveness, equity impact and quality of ANC services goes beyond the scope of this study. However, they are important considerations in any future efforts to scale-up ANC services in PNG and other LMICs. The results from this study, in particular the derivation of expected financial cost for ANC services enables additional research to address these key issues, which would complement our findings and the translation to policy and practice.

## Conclusion

Our findings suggest that significant additional investment in ANC services will be required to scale-up current ANC services to all pregnant women in PNG. Any contemplation of doubling the number of antenatal clinic visits to eight, as per the new WHO antenatal care guidelines, is perhaps not affordable with current GoPNG budget allocations to healthcare. Given this, considerations to invest in programmes to encourage early initiation of ANC during the first trimester may provide a cost-effective pathway to reduce adverse pregnancy and birth outcomes. The results from this study can be used as a benchmark for estimating health system costs and help policy makers in PNG, and other LMICs, strengthen their health systems through costing interventions in ANC to improve the quality of service delivery. Our findings may also inform future cost-effectiveness analyses of ANC policy reforms including, encouraging first-trimester initiation of ANC, or new interventions to expand access to quality ANC.

## supplementary material

10.1136/bmjopen-2023-080574 Supplementary file 1

10.1136/bmjopen-2023-080574 Supplementary file 2

10.1136/bmjopen-2023-080574 Supplementary file 3

10.1136/bmjopen-2023-080574 Supplementary file 4

10.1136/bmjopen-2023-080574 online supplemental file 5

## Data Availability

Data are available upon reasonable request. All data relevant to the study are included in the article or uploaded as supplementary information.
