# Supplementary material for: What does it cost to deliver antenatal care in Papua New Guinea? Results from a health system costing and budget impact analysis using cross-sectional data
Source: BMJ Open. 2024 Nov 27;14(11):e080574. doi: 10.1136/bmjopen-2023-080574 (PMC11603808; doi:10.1136/bmjopen-2023-080574)
Supplement: Supplementary file 1 [file bmjopen-14-11-s001.pdf]

Supplementary Appendix S1

WANTAIM Study Group

**Investigator Group**	
Andrew Vallely	Chief Investigator
William Pomat	Co-Chief Investigator
Michaela Riddell	Senior Trial Coordinator
Alice Mengi	Deputy Trial Coordinator
Steven Badman	Laboratory Coordinator
Lisa Vallely	Maternal and Child Health Coordinator
Handan Wand	Trial Statistician
Nicola Low	Epidemiologist
John Bolnga	Medical Expert - Madang
Delly Babona	Medical Expert – East New Britain
Glen Mola	Trial Medical Expert
Virginia Wiseman	Trial Health Economist
Angela Kelly-Hanku	Social Research Lead
Caroline Homer	Epidemiologist / Maternal and Child Health Lead
Christopher Morgan	Health Systems Research Lead
Stanley Luchters	Clinical Epidemiologist
David Whiley	Laboratory Research Lead
Leanne Robinson	Epidemiologist / Malaria Expert
Lucy Au	Site Coordinator – East New Britain
Irene Pukai Gani	Site Coordinator – East New Britain
Moses Laman	Clinical Epidemiologist / Malaria Expert
Grace Kariwiga	Trial Medical Expert
Pamela Toliman	Epidemiologist / Laboratory Researcher
Batura Neha	Health Economist
Sepehr Tabrizi	Laboratory Research Scientist
Stephen J Rogerson	Clinical Epidemiologist / Malaria Expert
Suzanne M Garland	Clinical Epidemiologist and Medical Expert
Rebecca Guy	Epidemiologist
Rosanna Peeling	Epidemiologist
John M Kaldor	Epidemiologist
**Trial Administration**	
Kelvin Waukave	Financial Controller
Wilfred Peter	Communications Advisor (Madang PHA)
Jacob Morewaya	Public Health Advisor (Milne Bay PHA)
Peter Siba	Senior Technical Advisor
Elizabeth Peach	Research Manager (Burnet Institute)
Patricia Sengele	Administration/Finance Officer (Burnet Institute)
**East New Britain Province**	
Leah Molok	HEO/Research Nurse

Tessie Clip	HEO/Research Nurse
Irene Daniels	HEO/USS technician
Crystal Keiwaga	HEO/USS technician
Daniel Hosea	Midwife/Research Nurse
Augustina Aiarak	Midwife/Research Nurse
Lorraine Mua	Midwife/Research Nurse
Valentine Russiat	Laboratory Officer/GeneXpert technician
Anna Davis	Research Nurse/GeneXpert technician
Biru Subey	Research Nurse/GeneXpert technician
Misilie Padik	Research Nurse
Muria Tangal	Research Nurse
Esleen Vovono	Research Nurse
Konsetta Malava	Research Nurse
Ellen Kavang	Research Nurse
Vicky Bayagau Wong	Research Nurse
Jermimah Garaen	Research CHW
Laniet Eddie	Research CHW
Johnslyne David	Research CHW
Jermimah Garaaen	Research CHW
Diana Malip	Research CHW
Noel Amada	Driver
Elisha Jordan	Driver
Mosely Viringa	Driver
Cosmos Francis	Driver (Burnet Institute)
James Makap	Driver (Burnet Institute)
Daniel Amin	Laboratory Officer (Burnet Institute)
Ruth Fidelis	Senior Laboratory Officer (Burnet Institute)
Tony Rave	HEO/Community Liaison Officer
Rebecca Anian	Community Liaison Officer/Admin Officer
Romalus Tavui	Community Liaison Officer
John Kamit	Community Liaison Officer
Benedictor Mission	Community Liaison Officer
Charity Stanley	Community Liaison Officer
Grace Baining	Finance officer (Burnet Institute)
Everlyn Kavang	HR officer (Burnet Institute)
**Madang staff**	
Sharon Warel	Midwife/Research Nurse/USS Technician
Janeth Kulimbao	Research CHW/USS Technician
Talitha Manie	Research Nurse /USS technician
Dupain Singirok	Snr Research Nurse
Carolyn Wokias Augusto	Snr Research Nurse/Community Liaison
Eunice Jally	HEO/Research Nurse
Maggie Taupa	Research Nurse
Regina Enman	Research Nurse
Judith Demie	Research Nurse

Pamela Brea	Research Nurse
Aileen Jeffrey	Research Nurse
Jonathon Warel	Research Nurse
Cornelia Duba	Laboratory Officer/GeneXpert Technician
George Kuias	Research Nurse/GeneXpert Technician
Beromina Jano	Research CHW/ GeneXpert Technician
Michelyn John	Research CHW/ GeneXpert Technician
Georgina Sengum	Research CHW
Joyce Soalili	Research CHW
Francesca Buran	Research CHW
Cegatha Tawai	Research CHW
Milda Lasu	Research CHW
Tia Marie Badem	Community Liaison Officer
Theresa Tivud	Community Liaison Officer
Joseph Yamuna	Operations Manager
Mathilda Saki	Data Manager
Kelly Masil	Data Manager
Lennie Mal	Data Entry Officer
Yapi Kepea	Driver
Paul Romanus	Driver
Konnie Patrick	Driver/Community Liaison Officer
**Study Sites**	
Paparatava Health Centre	Antenatal Clinic, Labour/Postnatal, East New Britain (CHS)
Kerevat Rural Hospital	Antenatal Clinic, Labour/Postnatal, East New Britain (PHA)
Warangoi Rural Hospital	Antenatal Clinic, Labour/Postnatal, East New Britain (PHA)
Gelegele Health Centre	Antenatal Clinic, Labour/Postnatal, East New Britain (PHA)
Napapar Health Centre	Antenatal Clinic, Labour/Postnatal, East New Britain (CHS)
St Mary’s Vunapope	Labour/Postnatal, East New Britain (CHS)
Nonga General Hospital	Labour/Postnatal, East New Britain (PHA)
Jomba Clinic	Antenatal Clinic, Madang (PHA)
Madang Town Clinic	Antenatal Clinic, Madang (PHA)
Alexishafen Health Centre	Antenatal Clinic, Labour/Postnatal, Madang (CHS)
Mugil Health Centre	Antenatal Clinic, Labour/Postnatal, Madang (CHS)
Yaguam Rural Hospital	Antenatal Clinic, Labour/Postnatal, Madang (LHS)
Madang Provincial Hospital	Labour/Postnatal, Madang (PHA)
