# Supplementary material for: What does it cost to deliver antenatal care in Papua New Guinea? Results from a health system costing and budget impact analysis using cross-sectional data
Source: BMJ Open. 2024 Nov 27;14(11):e080574. doi: 10.1136/bmjopen-2023-080574 (PMC11603808; doi:10.1136/bmjopen-2023-080574)
Supplement: Supplementary file 2 [file bmjopen-14-11-s002.pdf]

Supplementary Figure 1: Illustration of the PNG fANC model

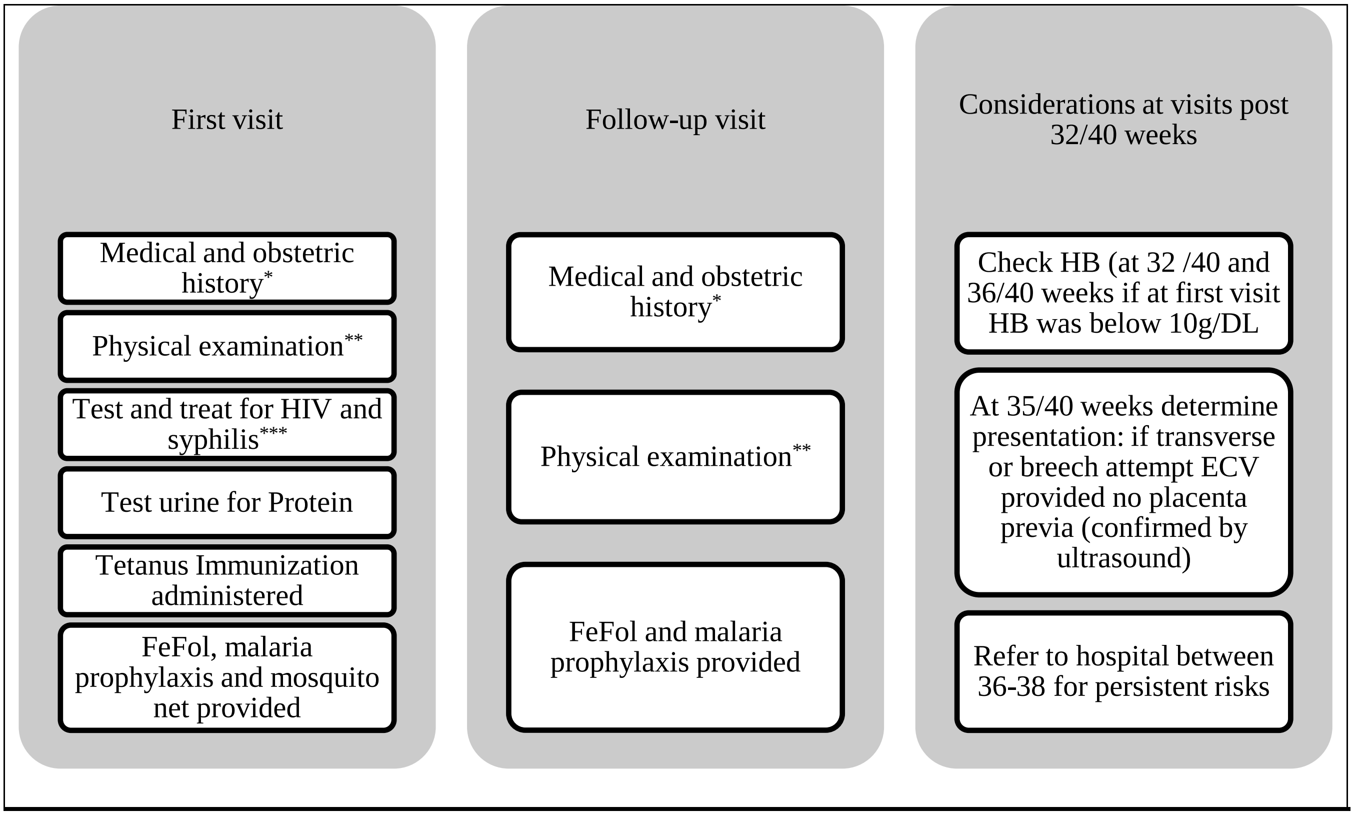

*Medical and obstetric history includes calculating EDD and EGA and discuss family planning.

**Physical examination incldues measuring fundal height, weight and taking blood pressure.

***HIV and syphilis testing and treatment includes pre- and post- test counselling.

Abbreviations: ECV: External cephalic version; EED: estimated date of delivery; EGA: estimated gestational age; FeFol: Ferrous Sulphate and Folic Acid; HB: haemogloblin; HIV: human immunodeficiency virus; malaria prophylaxis: sulfadoxine pryrimethamine/ Fansidar.
