# Supplementary material for: What does it cost to deliver antenatal care in Papua New Guinea? Results from a health system costing and budget impact analysis using cross-sectional data
Source: BMJ Open. 2024 Nov 27;14(11):e080574. doi: 10.1136/bmjopen-2023-080574 (PMC11603808; doi:10.1136/bmjopen-2023-080574)
Supplement: Supplementary file 3 [file bmjopen-14-11-s003.pdf]

Supplementary Table 1: Overview of the health facilities included in this study

	Management	Pregnant women in catchment* N (Range)	Antenatal clinic attendees* N (Range)	Clinicians providing ANC services** N (Range)
East New Britain (n=5)				
Level 4 (n=1)	Public	905	750	1
Level 3 (n=1)	Public	487	450	1
Level 2 (n=3)	Public (n=1) Church (n=2)	593 (451- 676)	433 (216- 604)	2 (1-2)
Madang (n=4)				
Level 4 (n=1)	Church	499	500	3
Level 3 (n=1)	Church	2273	508	2
Level 2 (n=2)	Public (n=2)	565***	619 (542-696)	4 (3-5)

*Annually reported number

**Does not include laboratory technicians or clinicians working in voluntary counselling and testing (VCT) who provide antenatal testing for HIV and syphilis

***Health facilities are in the same catchment area, therefore the number of births recorded is the same.
