# Supplementary material for: What does it cost to deliver antenatal care in Papua New Guinea? Results from a health system costing and budget impact analysis using cross-sectional data
Source: BMJ Open. 2024 Nov 27;14(11):e080574. doi: 10.1136/bmjopen-2023-080574 (PMC11603808; doi:10.1136/bmjopen-2023-080574)
Supplement: Supplementary file 4 [file bmjopen-14-11-s004.pdf]

Supplementary Table 1: Service statistics per province (2019)

Province	Total number of pregnancies (N)	N (%) of pregnant women attending antenatal clinic	
		At least once	At least four times
Manus	1843	1175 (63.8%)	808 (43.8%)
Gulf	8464	2727 (32.2%)	1126 (13.3%)
Oro	9576	2949 (30.8%)	1456 (15.2%)
New Ireland	7167	3847 (53.7%)	2289 31.9%)
Western	12146	3985 (32.8%)	1762 (14.5%)
Central	10431	4152 (39.8%	1638 (15.7%)
Jiwaka	11686	4782 (40.9%)	2193 (18.8%)
West Sepik	12834	4887 (38.1%)	2109 (16.4%)
Hela	10391	5229 (50.3%)	3203 (30.8%)
Chimbu	11137	5378 (48.3%)	2751 (24.7%)
Autonomous Region of Bougainville	12409	6016 (48.5%)	2763 (22.3%)
Milne Bay	12762	6851 (53.7%)	4171 (32.7%)
Southern Highlands	23789	7083 (29.8%)	3435 (14.4%)
West New Britain	12233	7108 (58.1%	3743 (30.6%)
Enga	15923	7303 (45.9%)	2298 (14.4%)
East Sepik	22476	7687 (34.2%)	2784 (12.4%)
East New Britain	13234	8158 (61.6%)	4176 (31.6%)
Western Highlands	14380	8546 (59.4%)	4295 (29.9%)
Madang	27403	10907 (39.8%)	5045 (18.4%)
Eastern Highlands	23147	12151 (52.5%)	6608 (28.5%)
Morobe	30746	14372 (46.7%)	5991 (19.5%)
National Capital District	26014	14560 (56.0%)	7531 (28.9%)
