# Supplementary material for: What does it cost to deliver antenatal care in Papua New Guinea? Results from a health system costing and budget impact analysis using cross-sectional data
Source: BMJ Open. 2024 Nov 27;14(11):e080574. doi: 10.1136/bmjopen-2023-080574 (PMC11603808; doi:10.1136/bmjopen-2023-080574)
Supplement: online supplemental file 5 [file bmjopen-14-11-s005.pdf]

Supplementary Appendix S2

Equations accompanying the Costing approach

Equations deriving the annual health system cost for antenatal care, the cost of scaling up to universal coverage and the expected cost of the eight-visit model	Equation Number	Notes
**Capital Costs:**		
$C = (E + I)$	EQ1	C: Capital costs;<br>E: Cost of equipment; and<br>I: Cost of infrastructure.
**Recurrent Costs for the first and follow-up antenatal clinic visits**		
$R_1 = (H + M_1 + O)$	EQ2a	H: Cost of human resources;<br>O: Cost of overheads;<br>M_1: Cost of medicines and medical Supplies used during the first visit; and<br>R_1: Recurrent costs for the first antenatal clinic visit
$R_2 = (H + M_2 + O)$	EQ2b	M_2: Cost of medicines and medical supplies used during a follow-up visit; and<br>R_2: Recurrent costs for the follow-up antenatal clinic visit
**Cost of the first and follow-up antenatal clinic visits**		
$ANC_1 = C + R_1$	EQ3a	$ANC_{1:}$ Cost of the first antenatal care visit
$ANC_2 = C + R_2$	EQ3b	$ANC_{2:}$ Cost of the follow-up antenatal care visit
**Cost of four antenatal clinic visits**		
$fANC = ANC_1 + 3(ANC_2)$	EQ4	fANC: Cost of four antenatal clinic visits (also called focused antenatal care)

Annual health system cost for antenatal care		
$fANC \times U$	EQ5	U: antenatal care utilization (number of pregnant women visiting antenatal clinic at least four times throughout pregnancy)

Expected cost of universal coverage of four antenatal clinic visits[*,**]		
$UC = \left(1(R_1) + 3(R_2)\right) \times P$	EQ6	UC: Expected financial cost of universal coverage of four antenatal clinic visits (or of focused antenatal care); P: population of pregnant women<br>*: only includes financial costs<br>**: costs are not discounted

Expected cost of universal coverage of eight antenatal clinic visits or budget impact analysis[*,**]		
$BIA = (1(R_1) + 7(R_2)) \times P$	EQ7	BIA: budget impact analysis of making eight antenatal clinic visits throughout pregnancy<br>*: only includes financial costs<br>**: costs are not discounted
