# Supplementary material for: What does it cost to deliver antenatal care in Papua New Guinea? Results from a health system costing and budget impact analysis using cross-sectional data
Source: BMJ Open. 2024 Nov 27;14(11):e080574. doi: 10.1136/bmjopen-2023-080574 (PMC11603808; doi:10.1136/bmjopen-2023-080574)
Supplement: Supplementary file 6 [file bmjopen-2023-080574.reviewer_comments.pdf]

PEER REVIEW HISTORY

BMJ Open publishes all reviews undertaken for accepted manuscripts. Reviewers are asked to complete a checklist review form and are provided with free text boxes to elaborate on their assessment. These free text comments are reproduced below.

ARTICLE DETAILS

Title (Provisional)

What does it cost to deliver antenatal care in Papua New Guinea? Results from a health system costing and budget impact analysis using cross sectional data.

Authors

Saweri, Olga phoebe martinella; Batura, Neha; Pomat, William; Vallely, Andrew John; Wiseman, Virginia

---

VERSION 1 - REVIEW

**Reviewer**	**1**
**Name**	**Babigumira, Joseph**
**Affiliation**	**GHE Consulting**
**Date**	**16-Nov-2023**
**COI**	**None**

November 16, 2023

Edit
or
BMJ
Ope
n

Review of manuscript BMJ Open 2023-080574 “What does it cost to deliver antenatal care in a low- and middle-income setting? Estimating health system costs and affordability in Papua New Guinea”

Dear Editor,

Thank you for the opportunity to review this manuscript. Please see below my comments to the authors with suggestions for possible changes to the manuscript.

Sincerely,

Joseph B. Babigumira

COMMENTS

1. Methods. The estimation of cost of scaling up of universal coverage of four antenatal clinics is unclear and likely overestimated. The cost of scaling up four visits needs to be adjusted for the proportion of women in PNG that attend 1, 2, and 3 visits. Additionally, scale up costs for these categories of women (including women that do not attend ANC) may include provider related costs of reaching women e.g., health education and communication.

2. Methods: The inclusion of a discount rate in the univariate sensitivity analysis table suggests that costs were discounted over a 5-year time horizon. Discounting is usually not recommended as the payer is interested in the budget impact and financial streams, undiscounted, at each point in time. What is the justification for discounting?

3. Methods: I would suggest that the inputs into the univariate sensitivity analysis should include resource use estimates (e.g., personnel time for clinic visits) and unit costs (e.g., time-specific salary). Why are the inputs into the univariate sensitivity analysis already aggregated e.g., personnel without consideration of resource use and unit cost?

4. Methods and results: The use of the phrase "unit costs" is confusing. Unit costs may refer to prices, which are multiplied by resource use estimates in micro-costing to estimate the cost of a given component of care e.g., personnel for ANC. You seem to use "unit costs" for the cost of antenatal visits (results) and "unit costs" for the prices of commodities in a micro-costing framework (e.g., under lines 187, 188, and 189).

5. Methods: It is not clear where top-down and bottom-up costing were used and for which components of the costs of an ANC visit. Clarifying this will make the paper easier to follow and understand.

6. General: The authors clearly state that they followed ISPOR guidelines for budget impact analysis. It might be worthwhile to make the use and consideration of this guidance more structured in the methods and reporting, including issues of anticipated uptake of new ANC services e.g., scale up from 4 to 8 visits; current mix of interventions (proportion of 0, 1, 2, and 3 ANC visits); and cost of these mixes. The authors also use the CHEERS framework which is probably more suited to full economic evaluations. This is admirable but a structured consideration of the BIA guidance is better suited to this study.

7. Discussion: Is scale up of 4 visits nationally or 8 visits cost-effective? It might enrich the paper if some discussion of the cost-effectiveness or potential cost-effectiveness of these interventions is considered. Budget impact is only relevant, in my view, if policy makers are considering a cost- effective intervention.

8. Minor: Examine and potentially amend the sentence transition (lines 128, 129, and 130).

9. Minor: Edit sentence starting on line 328.

**Reviewer**	**2**
**Name**	**Chowdhury, Akibul Islam**
**Affiliation**	
**Date**	**04-Jan-2024**

COI **N/A**

I would like to thankl the authors for evaluating the costing in ANC visits and adopting the new guidelines of WHO. This study helps to encourage many researchers to do future studies to reduce the maternal and child mor

****The reviewer provided a marked copy with additional comments. Please contact the publisher for full details.**

Reviewer **3**

Name **Kumar, Meghan**

Affiliation **London School of Hygiene and Tropical Medicine Faculty of Public Health and Policy**

Date **09-Feb-2024**

COI **None.**

Overall:

1. This isn't the first / only country to be grappling with if and how to adopt the 8 ANC visits recommendation. Drawing more upon that in the background would be helpful – I think less content on the global problem of maternal/newborn deaths and stillbirths generally (current paragraph 1) and more focus on introducing the motivation for the new 8 visit model early and describing the policy problem this engenders in PNG and elsewhere, then citing the literature where else there have been studies that looked at this. This will place the study more precisely from the outset

2. Really appreciate that you have a BIA – it's critical to think about budget impact not just in absolute value but in terms of the financing of the health sector in which you're working. So either as a percentage of general government expenditure for health and/or thinking about the role of donor financing etc to compare to total health expenditure per capita. This will give the policymaker and the reader an idea of whether mobilising these resources is reasonable. You cover some of this in the discussion but I think this belongs in the results section and then you can explore fiscal space, priority setting etc in the discussion instead.

Background:

• statement line 64 – 66: "improvements in coverage linked to adequate services." Consider clarifying whether adequate means high-quality or what is the link you are proposing between coverage and outcomes.

• Line 67: does adverse MNH mean mortality or do you include other outcomes here?

• Line 68-9: why such a huge range of MMR estimates?

• Line 71: relatively low compared to what? Are these in line with other countries in the region or at the same level of development?

• Line 80: suggest stating “recommended ANC visits” for clarity

• Line 93: you mention here (as well as in strengths section) that this is the second study assessing ANC costs. More than the number, it would be important to say for what country and what findings were – why this is relevant to your study or how you expect similarities/differences

Methods:

• Study setting:

o clarity on levels at which specific high-impact ANC interventions can be / are delivered would be helpful (eg HIV/anemia/malaria/syphilis testing, ultrasound), perhaps in Supp Fig 1

o You don’t describe anything in this section about the two different provinces where the facilities are located. Even if you think they are comparable in terms of key features, a nod to that in the text would be helpful to show you have considered it.

• What do you think the impact is of the limitations you listed – eg small sample size?

• Paragraph beginning line 128: suggest moving supp table 1 to the main text for simplicity. Clarify “monthly” in ANC attendees, add the number of maternity beds, be consistent with language (faith-based vs. church operated)

• Line 125: clarify what is routine ANC – is it fANC plus the PoC testing, and if so maybe list the infections included as well as whether this can be delivered at all levels of the health system in terms of HR, supplies

• Line 138-9: why 9 of 10 facilities?

• Any issues related to water mains and power supply/backup reliability that need accounting for?

• Line 198: are you suggesting that the costs are the same for public and FBO-operated clinics

Results:

• See overall comments on BIA and approach

• Figure 1:

o Is it worth presenting an overall / average for the country given that I think you’ll use that in the extension to other provinces and sites – or do you extend from an average at each level?

o What about putting HR as the bottom of the stacked bar given it’s by far the largest cost component in all sites?

• Figure 2:

o I'd consider the presenting both the total costs and also the per capita costs or budget impact % of financial spend if you are keeping this in the body

o Could consider dropping this to the appendix

• Figure 3:

o Rethink as you look at the BIA and what you want to include

• Figure 4:

o Could move to appendix and just describe which had the largest impact and then discuss how good you think your estimates are – HUGE impact of HR and ANC coverage that needs to come out clearly

Discussion:

• Suggest move the budget impact percentages into findings and spend more time on the feasibility of adopting any of these approaches including fiscal space, political economy, disinvestment considerations given the health financing of the sector as a whole

• Line 355: exuberated should read exacerbated

• I think spending some time in the discussion on the implications of scaling up to universal ANC or 8 ANC visits on equity and quality is key to the argument you're making. You touch on this in lines 377-78 but I think you could engage with this more seriously and consider eg whether targeted strategies for specific regions, population sub-groups might be more feasible and effective at reducing the mortality outcomes on which your study focuses.

• Line 386: you drop in 'and other LMICs' – please give some more space to why or why not these findings may be transferable to other contexts and what aspects of context are critical if policymakers in other countries would consider whether these findings are relevant

---

VERSION 1 - AUTHOR RESPONSE

20th July 2024

BMJ open

Manuscript Title: What does it cost to deliver antenatal care in a low- and middle-income setting? Estimating health system costs and the budget impact of antenatal care in Papua New Guinea

Authors: Olga PM Saweri, Neha Batura, William S Pomat, Andrew J Vallely, Virginia Wiseman

for the WANTAIM Study Group

Dear Editor,

I, on behalf of my co-authors, am pleased to submit a major revision to an original research manuscript for publication in BMJ open.

Antenatal care (ANC) is a cost-effective intervention which reduces maternal mortality through routine antenatal clinic visits. These visits assess the progression of pregnancy and ensure the health and well-being of both mother and baby. ANC is also critical to assess risks associated with adverse pregnancy and birth outcomes. Despite the importance of ANC services to maternal health, few studies have costed ANC services in low- and middle-income settings and none in Pacific. Papua New Guinea (PNG) has one of the highest maternal mortality ratios in the Asia-Pacific region and urgently requires evidence on the level of investment needed to expand access to antenatal services. This study addresses this evidence gap by comprehensively costing ANC services in PNG and by estimating the expected level of (financial) investment required to scale up to universal coverage of ANC services for four and eight visits.

Using detailed micro-costing and top-down approaches we costed ANC services from a provider perspective. Costs were collected from nine health facilities in two provinces as part of a randomized cross-over clinical trial (WANTAIM trial). The study illustrated that the annual health system cost was $6.9 million (K16.9 million), scale up to universal coverage of four visits cost $22.7 million (K55.2million), and adopting the eight-visit model had an annual average budget impact of $45.4 million (K110.3 million) over five years. Costs varied with the number of clinicians, infrastructure, and antenatal care coverage. The results of this study illustrate that ensuring universal access to the fANC model of ANC (based on 4 visits) would require substantial investment. Further, the methods utilised in this study can be replicated in other low- and middle- income country settings to calculate ANC service provider costs.

This re-submission includes a detailed response to all reviewer (and editorial) feedback in the 'response to reviewers' document, a marked copy of the manuscript and a clean version of the manuscript. I confirm that this original manuscript has not been published, wholly or in part, in any other journal. All authors of this publication have contributed to the manuscript, the revisions of the original submission, and have agreed to its re-submission to BMJ open. I also confirm that my co-authors and I have no conflicts of interest.

We look forward to hearing from you.

Best,

Olga PM Saweri

PhD Candidate

The Kirby Institute, (University of New South Wales) & Papua New Guinea Institute of Medical Research.

VERSION 2 - REVIEW

**Reviewer**	**1**
**Name**	**Babigumira, Joseph**
**Affiliation**	**GHE Consulting**
**Date**	**19-Aug-2024**
**COI**	**None.**

The response to reviewer comments was comprehensive and satisfactory.
